# Prognostic value of circulating tumor DNA in pancreatic cancer: a systematic review and meta-analysis

**DOI:** 10.18632/aging.202199

**Published:** 2020-12-09

**Authors:** Zengli Fang, Qingcai Meng, Bo Zhang, Si Shi, Jiang Liu, Chen Liang, Jie Hua, Xianjun Yu, Jin Xu, Wei Wang

**Affiliations:** 1 Department of Pancreatic Surgery, Fudan University Shanghai Cancer Center, Shanghai 200032, China; 2 Department of Oncology, Shanghai Medical College, Fudan University, Shanghai 200032, China; 3 Shanghai Pancreatic Cancer Institute, Shanghai 200032, China; 4 Pancreatic Cancer Institute, Fudan University, Shanghai 200032, China

**Keywords:** pancreatic cancer, circulating tumor DNA, prognosis, K-ras, systematic review and meta-analysis

## Abstract

Increasing evidence has revealed the potential correlation between circulating tumor DNA (ctDNA) and the prognosis of pancreatic cancer, but inconsistent findings have been reported. Therefore, a meta-analysis was performed to evaluate the prognostic value of ctDNA in pancreatic cancer. The Embase, MEDLINE, and Web of Science databases were searched for relevant articles published until April 2020. Articles reporting the correlation between ctDNA and the prognosis of pancreatic cancer were identified through database searches. The pooled hazard ratios (HRs) for prognostic data were calculated and analyzed using Stata software. A total of 2326 patients pooled from 25 eligible studies were included in the meta-analysis to evaluate the prognostic value of ctDNA in pancreatic cancer. Patients with mutations detected or high concentrations of ctDNA had a significantly poorer overall survival (OS) (univariate: HR = 2.54; 95% CI, 2.05-3.14; multivariate: HR = 2.07; 95% CI, 1.69-2.54) and progression-free survival (PFS) (univariate: HR = 2.18; 95% CI, 1.41-3.37; multivariate: HR = 2.20; 95% CI, 1.38-3.52). In conclusion, the present meta-analysis indicates that mutations detected or high concentrations of ctDNA are significant predictors of OS and PFS in patients with pancreatic cancer.

## INTRODUCTION

Pancreatic cancer (PC) is one of the most lethal malignancies [1], with 47,050 estimated deaths in the United States in 2020 [2]. It is predicted to become the second leading cause of cancer-related deaths within the next decade, while the mortalities of other gastrointestinal cancers have declined over time [2, 3]. The poor outcomes of PC patients are attributed to diagnostic difficulty, early metastatic tendency and therapy resistance [4, 5]. Although the diagnostic and therapeutic strategies are constantly advancing, the mortality of PC patients remains high. Hence, for PC patients, valid biomarkers with simple test methods are urgently needed to predict survival outcomes, monitor disease changes, assess treatment responses and optimize treatment plans.

Liquid biopsy is a new technology of noninvasive detection that obtains biomarkers from nonsolid biological tissues such as blood [6], and it has received increasing attention in cancer research due to its convenience and noninvasiveness [7, 8]. Through the analysis of obtained markers, information from the source tissues can be reflected and cancers or other diseases can be monitored. For example, several recent studies have successfully applied liquid biopsy to achieve diagnosis and staging of PC [9, 10]. One of the biomarkers of liquid biopsy, circulating tumor DNA (ctDNA) arises from the apoptosis, necrosis, and lysis of circulating tumor cells, as well as active secretion from tumors [11, 12]. With the improvement of detection methods, ctDNA has been proposed as a biomarker for early diagnosis, prognostic stratification, disease monitoring and treatment response assessment in a variety of cancers [6, 13–15].

A growing body of studies has revealed the potential prognostic value of ctDNA in PC in recent years [16, 17]. The mutations and concentration levels of ctDNA may be associated with overall survival (OS) and progression-free survival (PFS) in PC patients. Our previous studies demonstrated that *K-ras* mutations and *ERBB2* exon17 mutations detected in ctDNA are significantly associated with OS [18–20]. However, inconsistent findings have been reported [21, 22]. Systematic and significant evidence that validates the prognostic value of ctDNA in PC is lacking. Therefore, the present systematic review and meta-analysis was performed to clarify the relationship between ctDNA and the prognosis of PC patients to provide more reliable evidence that may improve the management and treatment of PC.

## RESULTS

### Literature screening and study characteristics

**Figure 1:**
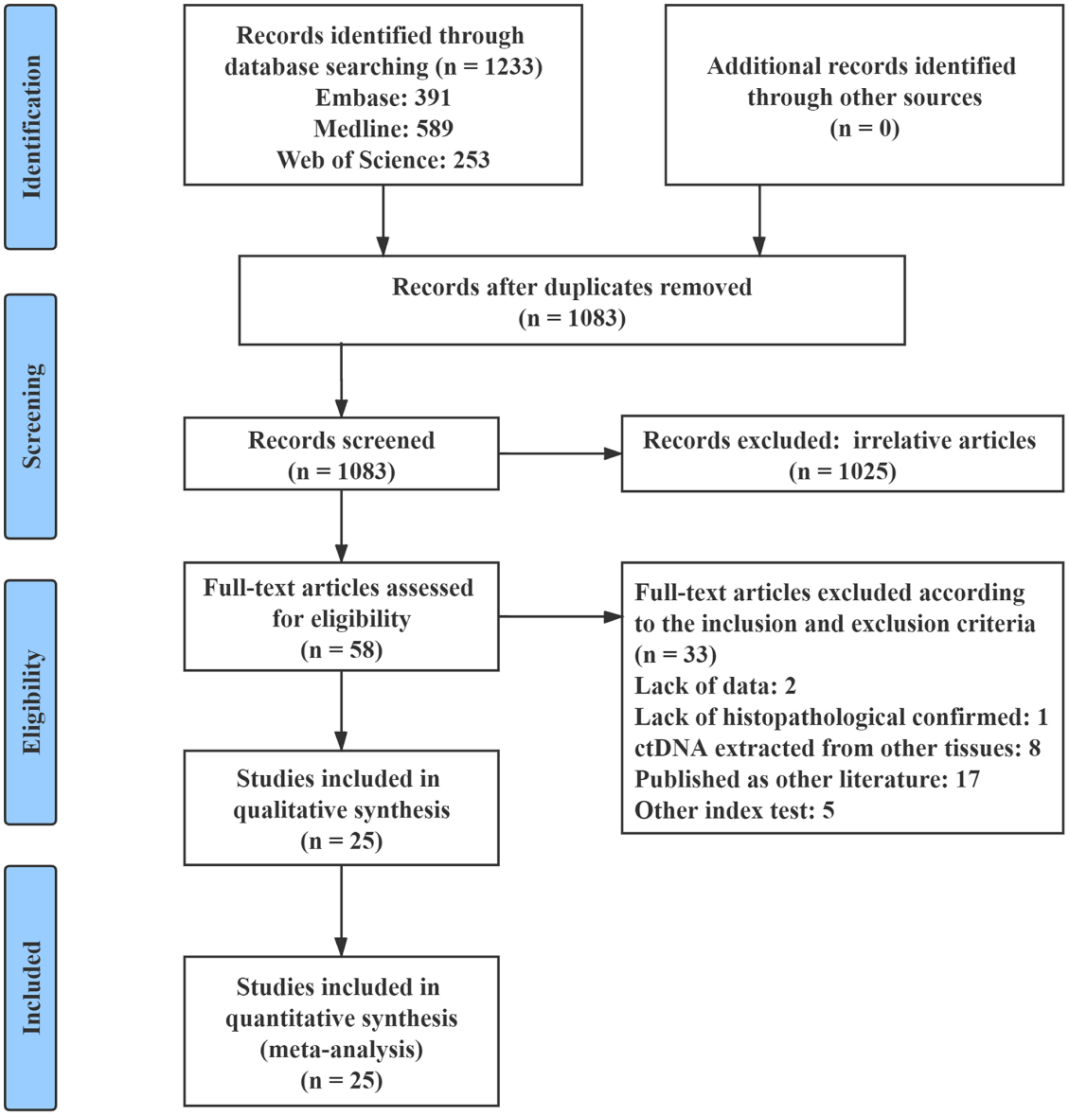
**Flow chart of the study selection process.**

The flow chart of the study selection criteria is presented in Figure 1. We initially identified 1233 studies from the Embase, MEDLINE, and Web of Science databases. Among these studies, 1175 were excluded after screening the titles and abstracts to remove duplicates or irrelevant studies. A total of 33 studies were excluded according to the inclusion and exclusion criteria. Thus, a total of 25 eligible studies that included 2326 patients were enrolled in this meta-analysis [18–42]. The characteristics of the included studies are summarized in Table 1. All of the included studies had OS outcomes, while 5 of them had PFS prognostic data [21, 28, 32, 35, 36]. Seven studies included patients with PC of all stages (stage: I - IV) [23–26, 29, 32, 33], 8 studies included patients with resectable PC (stage: I - II) [21, 22, 27, 31, 35, 37, 38, 42], and 12 studies included patients with advanced PC (stage: III - IV) [18–20, 22, 28, 30, 31, 34, 36, 39–41]. All of the included studies sampled at baseline, and 4 of them had postoperative data [21, 31, 35, 38]. For the quality assessment according to the Newcastle-Ottawa Scale (NOS), all of the included studies obtained a score greater than 6 (Supplementary Table 1).

**Table 1:** Characteristics of the Included Studies.

**Study**	**Country**	**No. of Patients**	**Tumor Stage**	**Sample**	**Sampling Time**	**ctDNA(+) Rate**	**Method**	**Marker**	**Survival Analysis**	**Result**
Castells 1999 [23]	Spain	44	I - IV	Plasma	Baseline	12/44, 27%	RFLP-PCR	*K-ras*	U/M	OS
Chen 2010 [18]	China	91	III - IV	Plasma	Baseline	30/91, 33%	PCR	*K-ras*	U/M	OS
Earl 2015 [24]	Spain	31	I - IV	Plasma	Baseline	8/31, 26%	ddPCR	*K-ras*	U/M	OS
Kinugasa 2015 [25]	Japan	75	I - IV	Serum	Baseline	47/75, 63%	ddPCR	*K-ras* / G12V/ G12D	U/M	OS
		66				36/66, 55%				
Takai 2015 [26]	Japan	259	I - IV	Plasma	Baseline	83/259, 32%	ddPCR	*K-ras*	M	OS
Hadano 2016 [27]	Japan	105	I - II	Plasma	Baseline	33/105, 31%	ddPCR	*K-ras*	U/M	OS
Tjensvoll 2016 [28]	Norway	14	III - IV	Plasma	Baseline	10/14, 71%	PNA-clamp PCR	*K-ras* / ctDNA level	U/M	OS / PFS
Adamo 2017 [29]	UK	26	I - IV	Plasma	Baseline	7/26, 27%	Targeted-NGS	*K-ras*	U	OS
Chen 2017 [30]	Denmark	189	III - IV	Plasma	Baseline	177/189, 94%	PCR coupled with NGS	ctDNA level	U/M	OS
Cheng 2017 [19]	China	188	III - IV	Plasma	Baseline	25/188, 13%	ddPCR	ERBB2 exon17	U/M	OS
						65/188, 35%		*K-ras* G12V		
Pietrasz 2017 [31]	France	104	III - IV	Plasma	Baseline	50/104, 48%	NGS	Multi-genes	U/M	OS
		31	I - II		Post-operative	6/31, 19%				
Kim 2018 [32]	Korea	106	I - IV	Plasma	Baseline	53/106, 50%	ddPCR	*K-ras*	U/M	OS / PFS
Lin 2018 [33]	China	65	I - IV	Plasma	Baseline	20/65, 31%	ddPCR	*K-ras*	U/M	OS
Nakano 2018 [21]	Japan	45	I - II	Serum	Baseline	11/45, 24%	PNA-clamp PCR	*K-ras*	U	OS / PFS
					Post-operative	20/45, 44%				
Perets 2018 [34]	Israel	17	III - IV	Plasma	Baseline	5/17, 29%	PCR	*K-ras*	U	OS
Yang 2018 [35]	China	35	I - II	Plasma	Baseline	9/35, 26%	dPCR	*K-ras* G12V	U/M	OS / PFS
					Post-operative	8/35, 23%				
Bernard 2019 [36]	USA	102	III - IV	Plasma	Baseline	53/102, 52%	ddPCR	*K-ras*	U/M	OS / PFS
Groot 2019 [37]	USA	59	I - II	Plasma	Baseline	29/59, 49%	ddPCR	*K-ras*	U/M	OS
Lee 2019 [38]	Australia	37	I - II	Plasma	Baseline	23/37, 62%	Safe-Sequencing System	*K-ras*	U/M	OS
		35			Post-operative	13/35, 37%				
Mohan 2019 [39]	UK	55	III - IV	Plasma	Baseline	40/55, 73%	ddPCR	*K-ras*	U	OS
Patel 2019 [40]	USA	94	III - IV	Plasma	Baseline	70/94, 74%	NGS	*K-ras* / ctDNA level	U/M	OS
Strijker 2019 [41]	Netherlands	58	III - IV	Plasma	Baseline	26/58, 45%	Targeted-NGS	ctDNA level	U/M	OS
Watanabe 2019 [22]	Japan	39	I - II	Plasma	Baseline	7/39, 20%	ddPCR	*K-ras*	U/M	OS
		39	III - IV			12/39, 31%				
Cheng 2020 [20]	China	210	III - IV	Plasma	Baseline	61/210, 29%	ddPCR	*K-ras* G12V	U/M	OS
						93/210, 44%		*K-ras* G12D		
Guo 2020 [42]	China	113	I - II	Plasma	Baseline	26/113, 23%	ddPCR	*K-ras* G12V	U/M	OS
						13/113, 12%		*K-ras* G12D		

PCR: polymerase chain reaction; NGS: next-generation sequencing; RFLP-PCR: restriction fragment length polymorphism-PCR; dPCR: digital PCR; ddPCR: droplet digital PCR; PNA-clamp PCR: peptide nucleic acid-clamp PCR; OS: overall survival; PFS: progression-free survival; U: univariate analysis; M: multivariate analysis.

### ctDNA as a prognostic factor of PC

**Figure 2:**
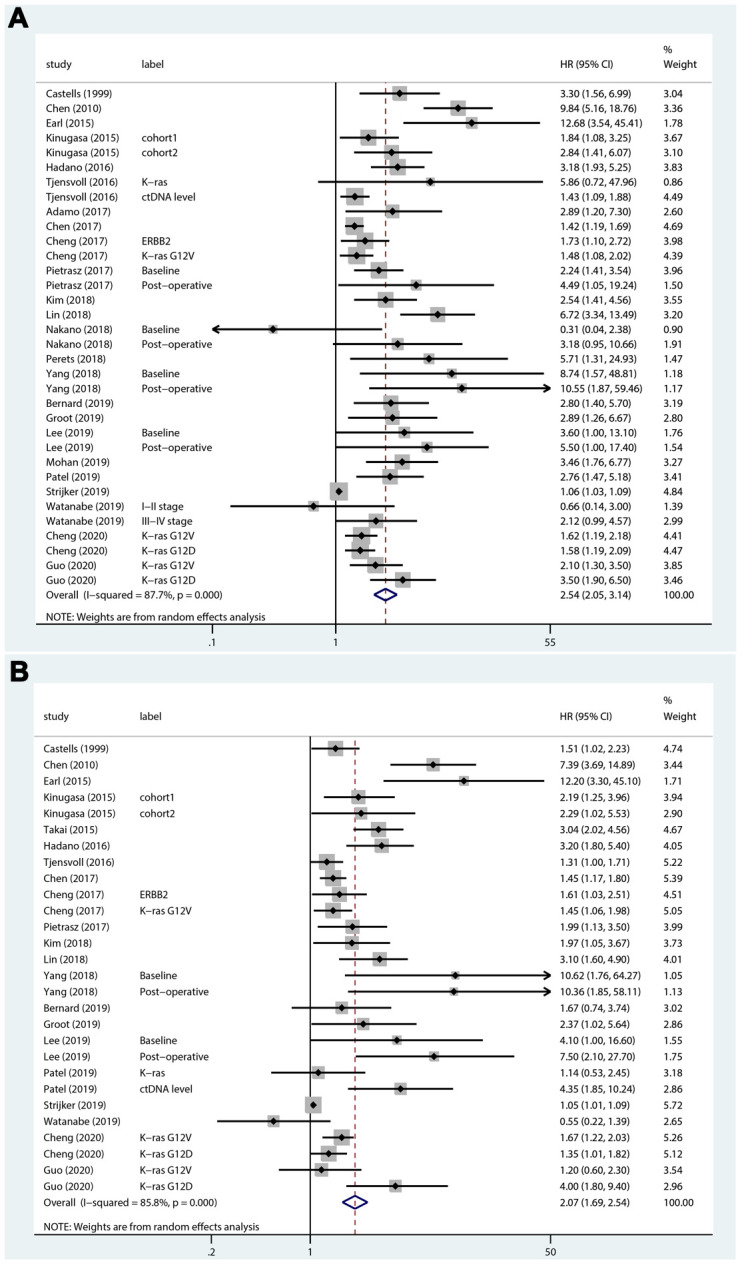
**Forest plots of the HRs for ctDNA and the OS of PC patients.** (**A**) The original HRs of OS analyzed by univariate analysis. (**B**) The original HRs of OS analyzed by multivariate analysis.

**Figure 3:**
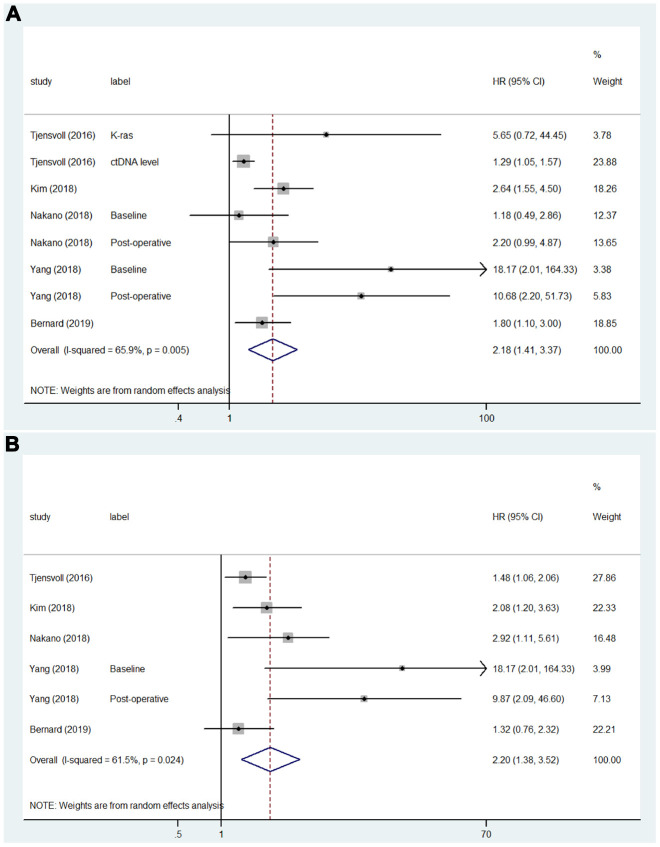
**Forest plots of the HRs for ctDNA and the PFS of PC patients.** (**A**) The original HRs of PFS analyzed by univariate analysis. (**B**) The original HRs of PFS analyzed by multivariate analysis.

A total of 24 studies that included 34 sets of samples were available for OS analyzed by univariate analysis and were pooled for meta-analysis. The meta-analysis results for ctDNA and the prognosis of PC patients are shown in Figure 2A. The results indicated that patients with mutations detected and high concentrations of ctDNA had a significantly poorer OS (HR = 2.54; 95% CI, 2.05-3.14). As shown in Figure 2B, for the multivariate analysis, 21 studies that included 28 sets of samples were analyzed and showed significant results (HR = 2.07; 95% CI, 1.69-2.54). Because of the significant heterogeneity (I^2^ = 87.7% and I^2^ = 85.8%), a random-effects model was applied. As shown in Figure 3A, for PFS, 5 studies that included 8 sets of samples were analyzed via univariate analysis, and the results showed significant associations between mutations detected and high concentrations of ctDNA and the PFS of PC patients (HR = 2.18; 95% CI, 1.41-3.37, I^2^ = 65.9%). Similar results (Figure 3B) were found when analyzed by multivariate analysis (HR = 2.20; 95% CI, 1.38-3.52, I^2^ = 61.5%).

### Subgroup analysis

Subgroup analyses were performed because of the significant heterogeneity in the above results. As shown in Supplementary Figure 1A, in the subgroup analysis by disease stage, whether the studies included patients with PC of all stages (HR = 3.37; 95% CI, 2.22-5.12, I^2^ = 55.2%), resectable PC only (HR = 2.95; 95% CI, 2.11-4.12, I^2^ = 28.0%), or advanced PC only (HR = 2.01; 95% CI, 1.58-2.57, I^2^ = 89.0%), mutations detected and high concentrations of ctDNA were still significant predictors of poorer OS and presented a similar effect. When classified by the sampling time, as shown in Supplementary Figure 1B, the studies sampled postoperatively (HR = 4.84; 95% CI, 2.38-9.85, I^2^ = 0.0%) had higher HRs than the studies sampled at baseline (HR = 2.42; 95% CI, 1.95-3.01, I^2^ = 88.4%). For subgroup analysis by detection marker (Supplementary Figure 1C), the studies detected *K-ras* mutations (HR = 3.31; 95% CI, 2.53-4.34, I^2^ = 48.6%), *K-ras* G12V mutation (HR = 2.03; 95% CI, 1.51-2.74, I^2^ = 49.3%) or *K-ras* G12D mutation (HR = 1.77; 95% CI, 1.22-2.58, I^2^ = 55.6%) showed that mutations detected in ctDNA were significantly associated with poorer OS, and the concentration level of ctDNA was also significantly associated with OS (HR = 1.40; 95% CI, 1.07-1.83, I^2^ = 87.2%). Subgroup analyses were also performed according to the detection method, ctDNA positive rate, sample origin, patient ethnicity and patient size, and the results are summarized in Table 2.

**Table 2:** Subgroup analysis for overall survival.

**Subgroups**	**No. of Studies**	**HR**	**95% CI**	**I^2^**
Tumor Stage				
*I-IV*	7	3.37	2.22-5.12	55.2%
*I-II*	12	2.95	2.11-4.12	28.0%
*III-IV*	15	2.01	1.58-2.57	89.0%
Sampling Time				
*Baseline*	30	2.42	1.95-3.01	88.4%
*Post-operative*	4	4.84	2.38-9.85	0.0%
Detection Marker				
*K-ras*	20	3.31	2.53-4.34	48.6%
*K-ras G12V*	7	2.03	1.51-2.74	49.3%
*K-ras G12D*	4	1.77	1.22-2.58	55.6%
*ERBB2 exon17*	1	1.73	1.10-2.72	0.0%
*ctDNA level*	4	1.40	1.07-1.83	87.2%
Detection Method				
*PCR*	9	3.77	1.77-8.02	81.6%
*ddPCR*	17	2.36	1.90-2.94	62.3%
*NGS*	5	2.14	1.17-3.91	85.4%
*Safe-Sequencing System*	2	4.35	1.67-11.32	0.0%
*PCR&NGS*	1	1.42	1.19-1.69	0.0%
ctDNA(+) Rate				
*<40%*	19	3.08	2.22-4.28	74.1%
*>40%*	15	2.01	1.58-2.55	84.6%
Sample Origin				
*Plasma*	30	2.60	2.08-3.26	88.6%
*Serum*	4	2.05	1.16-3.65	36.1%
Ethnicity				
*Asian*	18	2.49	1.90-3.28	73.2%
*Caucasian*	16	2.47	1.85-3.30	85.5%
Patient Size				
*<50*	15	3.14	2.01-4.90	60.3%
*50-100*	8	3.10	1.58-6.09	93.6%
*>100*	11	1.91	1.60-2.27	55.1%

### Publication bias

**Figure 4:**
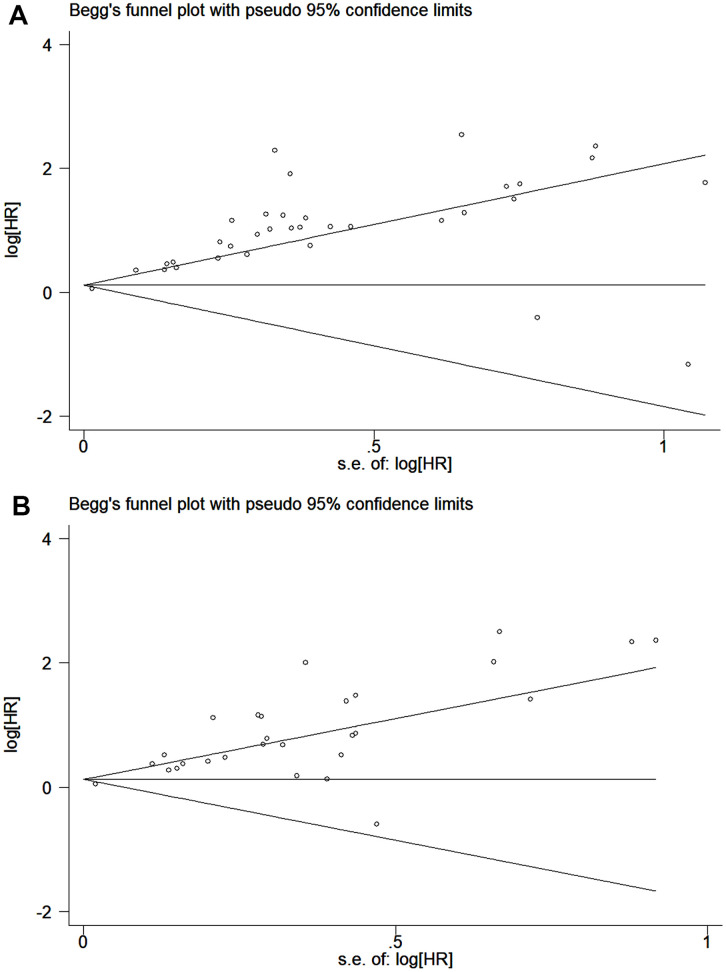
**Funnel plot for the assessment of publication bias of the included studies.** (**A**) The original HRs of OS analyzed by univariate analysis. (**B**) The original HRs of OS analyzed by multivariate analysis.

**Figure 5:**
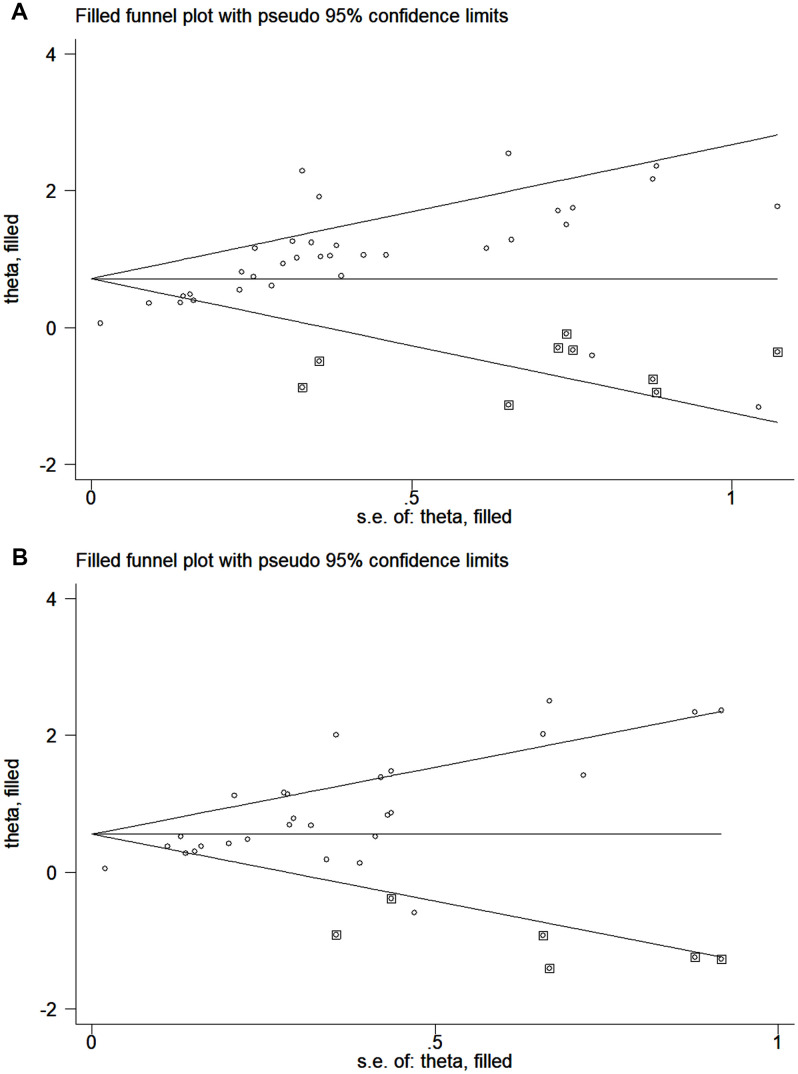
**Funnel plot for the trim and fill analysis.** (**A**) The original HRs of OS analyzed by univariate analysis. (**B**) The original HRs of OS analyzed by multivariate analysis.

The publication bias of the included studies was assessed by Egger’s and Begg’s tests in this meta-analysis. Significant publication bias was found in both the univariate and multivariate OS analyses according to the results of Egger’s and Begg’s tests (Figure 4A, 4B). Thus, we performed a trim and fill analysis, and the pooled HRs calculated by the trim and fill analysis still showed a significant effect of ctDNA in both the univariate OS analysis (HR = 2.05; 95% CI, 1.68-2.49) and multivariate OS analysis (HR = 1.74; 95% CI, 1.43-2.12). The funnel plot for the trim and fill analysis is shown in Figure 5A, 5B.

## DISCUSSION

PC is well known to have a poor prognosis, of which the five-year survival rate is approximately 10%, and only 15–25% of PC patients are resectable [4, 43]. Patients in different stages are suggested to undergo different treatments. For advanced PC, patients are usually suggested to undergo chemotherapy with combination regimens, but therapy resistance is a characteristic of PC [44, 45]. For resectable PC, extensive surgery with adjunctive chemotherapy is recommended; however, many resectable PC patients experience recurrence [46]. Thus, many studies have largely focused on finding effective prognostic factors. Increasing evidence has revealed the potential prognostic value of ctDNA in PC, although many studies validated their results using small sample sizes. However, inconsistent findings have been reported. Therefore, this systematic review and meta-analysis was performed to assess the potential prognostic value of ctDNA in PC.

A total of 2326 patients pooled from 25 eligible studies were included in this meta-analysis. The sample sizes for each study ranged from 14 to 259. The ctDNA was detected via different markers in these studies, which can be divided into two categories: mutations and concentration levels. Our study demonstrated that patients with mutations detected and high concentrations of ctDNA had significantly poorer OS (univariate: HR = 2.54; 95% CI, 2.05-3.14; multivariate: HR = 2.07; 95% CI, 1.69-2.54) and PFS (univariate: HR = 2.18; 95% CI, 1.41-3.37; multivariate: HR = 2.20; 95% CI, 1.38-3.52). Due to the significant heterogeneity, we performed subgroup analyses by tumor stage, sampling time, detection marker, etc. Significant differences were not observed between the results according to tumor stage and detection marker in the subgroup analyses. For the sampling time, the results showed that the studies that sampled postoperatively (HR = 4.84; 95% CI, 2.38-9.85, I^2^ = 0.0%) had higher HRs than the studies that sampled at baseline (HR = 2.42; 95% CI, 1.95-3.01, I^2^ = 88.4%). In terms of heterogeneity, the I^2^ value decreased to approximately 50.0% in most subgroups, which indicated that some of the sources of heterogeneity were the differences in tumor stages, sampling times, and detection markers.

As shown in the results of the subgroup analysis by detection methods, the I^2^ value decreased to approximately 60% in the droplet digital PCR (ddPCR) subgroups, which indicated that the differences in detection methods might be another potential source of heterogeneity. The technologies for ctDNA analysis are based on next-generation sequencing (NGS) or polymerase chain reaction (PCR). The methods based on PCR, such as digital PCR (dPCR), ddPCR and peptide nucleic acid-clamp PCR (PNA-clamp PCR), are more sensitive, quick, and inexpensive, but they can analyze only some specific loci once a time [14]. The methods based on NGS can analyze more loci and even perform whole-exome sequencing, but their sensitivity is lower than that of PCR-based methods. Thus, some researchers coupled the two technologies to reduce this limitation [30]. Before detection, the procedures of sample collection and ctDNA extraction are equally important [47]. However, standardized protocols for the collection, extraction and detection of ctDNA are lacking, which is one of the most important reasons why ctDNA has not been widely used in the clinic.

Most of the studies included in this meta-analysis focused on the mutation detection of ctDNA, especially *K-ras* mutations. *K-ras*, which encodes a small GTPase that modulates downstream signaling from growth factor receptors, is one of the major driver genes in PC and plays an important role in tumor progression [48]. Although the work of Nakano et al. [21] and Watanabe et al. [22] reported inconsistent findings, our analysis still shows that the detection of *K-ras* mutations in ctDNA had significant prognostic value in PC. In our view, we speculate that the reasons for the inconsistent results may be related to the use of different sources of samples, including plasma and serum; the inclusion of patients with pancreatic cancer in different stages that have received neo-adjuvant chemoradiotherapy or not; and the inclusion of patients with demographic differences. Several studies have reported that a type of *K-ras* mutations, the *K-ras* G12V mutation, is an independent prognostic factor in PC [19, 25, 35]. Cheng et al. [20] and Guo et al. [42] showed that the *K-ras* G12D mutation also has a significant prognostic value, although Kinugasa et al. [25] suggested that there is no significant association between the *K-ras* G12D mutation and OS in PC patients. According to the results of this meta-analysis, we considered that the detection of the *K-ras* G12D mutation is a significant prognostic factor in PC (HR = 1.77; 95% CI, 1.22-2.58, I^2^ = 55.6%), although more studies are needed to further confirm this finding. On the other hand, 4 studies focused on the concentration level of ctDNA [28, 30, 40, 41]. Although only 4 studies were included in this subgroup analysis, the heterogeneity among these studies was significantly high. We hold the opinion that in terms of concentration detection, different detection methods and cut-off values will have a greater impact on the results, which leads to high heterogeneity. Thus, more high-quality studies are needed to test the prognostic value of the concentration levels of ctDNA. In addition to these studies, Henriksen et al. reported that cell-free DNA promoter hypermethylation in plasma was associated with poorer OS [49]. We did not include this study in the meta-analysis because it studied cell-free DNA instead of ctDNA, and the detection methods between hypermethylation and mutation were considerably different. However, this study offers a potential idea and basis for further research on ctDNA.

In addition to one-point assessments of ctDNA, Watanabe et al. revealed that the longitudinal monitoring of ctDNA can also predict the prognosis of PC [22]. The emergence of ctDNA in longitudinal tests was associated with OS in both patients who underwent surgery (HR = 54.50; 95% CI, 6.64-447.60) and those who did not undergo surgery (HR = 10.40; 95% CI, 2.95-37.00). One of our recent studies reported that the detection of the *K-ras* G12V mutation in ctDNA correlated with circulating Treg cells can further stratify the prognosis in patients with advanced PC [20]. Patients with both the *K-ras* G12V mutation and high Treg cell levels had the worst survival. These studies suggested the great potential value in the innovative application of ctDNA for analyzing prognosis.

The prognostic value of ctDNA is not limited to OS and PFS. Lee et al. reported that ctDNA is associated with recurrence-free survival (RFS) [38]. For patients with resectable PC, postoperative ctDNA detection is a significant predictor of recurrence (HR = 6.30; 95% CI, 2.40-16.20). Guo et al. also found that the detection of the *K-ras* G12D in ctDNA is an independent prognostic factor of recurrence (HR = 5.10; 95% CI, 2.20-11.80) [42]. The effective prediction of recurrence plays an important guiding role in the selection of clinical treatment strategies, which allows for more aggressive screening and treatment to prevent recurrence.

Several limitations in the current meta-analysis should be considered. First, the methodological and clinical characteristics of the eligible studies were not quite consistent, which may cause heterogeneity in the overall results. Second, the differences in patient characteristics (such as age or other variables) may be another potential source of heterogeneity. Third, only studies published in English were included in our meta-analysis, which may result in bias. Finally, significant publication bias was observed in our meta-analysis. The results of the trim and fill analysis revealed that unpublished studies with negative conclusions might not impact the results of our meta-analysis. However, to provide more reliable evidence, the findings of our meta-analysis need to be confirmed by further multicenter, prospective clinical trials.

## CONCLUSIONS

In conclusion, the current meta-analysis indicates that there is a significant association between detectable ctDNA and PC prognosis. Mutations detected or high concentrations of ctDNA are significant predictors of the OS and PFS of PC patients. Our meta-analysis strongly supports that ctDNA is a useful predictive biomarker for PC, with advantages of convenience, safety and noninvasiveness.

## MATERIALS AND METHODS

### Search strategy

A systematic literature review was conducted by searching Embase, MEDLINE, and Web of Science from inception to April 2020. The keywords used in our searches were “circulating tumor DNA/ctDNA/cell free DNA/cfDNA”, “pancreatic cancer/pancreatic neoplasm/pancreatic ductal adenocarcinoma/PDAC” and “prognosis/prognostic”. The reference lists of the selected articles were also used to supplement the searches. Two reviewers (ZL Fang and QC Meng) independently assessed the eligibility of the potential studies following the guidelines of the Preferred Reporting Items for Systematic Reviews and Meta-Analyses (PRISMA) statement [50].

### Inclusion and exclusion criteria

The inclusion criteria were as follows: (1) all PC patients included in the studies were confirmed by histopathology; (2) ctDNA was extracted from peripheral blood; (3) the time of sample collection was declared; and (4) the prognostic value of ctDNA was reported or able to be calculated. The exclusion criteria were as follows: (1) ctDNA was not extracted from peripheral blood; (2) the HRs of prognostic data were not reported and Kaplan-Meier curves were not provided to calculate them; and (3) literature published as reviews, conferences, case reports, letters or expert opinions.

### Data extraction

The data were reviewed and extracted independently by two reviewers, and any disagreements were settled by discussion. The following information was extracted from the included articles: name of the first author, publication year, country of study, number of patients, tumor stage, sample origin, sampling time, ctDNA positive rate, detection method, detection marker, and HRs of prognostic data including OS and PFS. For articles whose HRs and 95% CIs were not directly available, we extracted the data from Kaplan-Meier curves via Engauge Digitizer version 4.1 [51] and used the methods described by Tierney et al. to calculate the HRs with 95% CIs [52].

### Quality assessment

The NOS was used to evaluate the quality of the included studies by two reviewers independently [53]. This scale consists of 8 items and is used to assess the quality of the selection, comparability, exposure (case-control studies) and outcomes (cohort studies) of the included studies. In the present study, we considered a study with a score of 6 or more as a high-quality study.

### Statistical analysis

Pooled HRs of OS and PFS were calculated using Stata 12.0 (StataCorp, College Station, TX, USA). If the original HRs were analyzed by both univariate and multivariate analyses, the pooled HRs were calculated respectively. Heterogeneity was assessed by chi-square tests and I^2^ values. For values of I^2^ > 50% or p < 0.1, the random-effects model was used; otherwise, the fixed-effects model was applied. All p-values were two sided, and statistical significance was set at p < 0.05. Subgroup analyses were performed according to the tumor stage, sampling time, detection marker, detection method, ctDNA positive rate, sample origin, ethnicity and patient size.

## Supplementary Material

Supplementary Figure 1

Supplementary Table 1

## References

[1] Shi S, Hua J, Liang C, Meng Q, Liang D, Xu J, Ni Q, Yu X. Proposed modification of the 8^th^ edition of the AJCC staging system for pancreatic ductal adenocarcinoma. Ann Surg. 2019; 269:944–50. 10.1097/SLA.0000000000002668 29334560

[2] Siegel RL, Miller KD, Jemal A. Cancer statistics, 2020. CA Cancer J Clin. 2020; 70:7–30. 10.3322/caac.21590 31912902

[3] Rahib L, Smith BD, Aizenberg R, Rosenzweig AB, Fleshman JM, Matrisian LM. Projecting cancer incidence and deaths to 2030: the unexpected burden of thyroid, liver, and pancreas cancers in the United States. Cancer Res. 2014; 74:2913–21. 10.1158/0008-5472.CAN-14-0155 24840647

[4] Hidalgo M. Pancreatic cancer. N Engl J Med. 2010; 362:1605–17. 10.1056/NEJMra0901557 20427809

[5] Wolfgang CL, Herman JM, Laheru DA, Klein AP, Erdek MA, Fishman EK, Hruban RH. Recent progress in pancreatic cancer. CA Cancer J Clin. 2013; 63:318–48. 10.3322/caac.21190 23856911 PMC3769458

[6] Crowley E, Di Nicolantonio F, Loupakis F, Bardelli A. Liquid biopsy: monitoring cancer-genetics in the blood. Nat Rev Clin Oncol. 2013; 10:472–84. 10.1038/nrclinonc.2013.110 23836314

[7] Marrugo-Ramírez J, Mir M, Samitier J. Blood-based cancer biomarkers in liquid biopsy: a promising non-invasive alternative to tissue biopsy. Int J Mol Sci. 2018; 19:2877. 10.3390/ijms19102877 30248975 PMC6213360

[8] Husain H, Velculescu VE. Cancer DNA in the circulation: the liquid biopsy. JAMA. 2017; 318:1272–74. 10.1001/jama.2017.12131 28973237 PMC5819336

[9] Yang KS, Im H, Hong S, Pergolini I, Del Castillo AF, Wang R, Clardy S, Huang CH, Pille C, Ferrone S, Yang R, Castro CM, Lee H, et al. Multiparametric plasma EV profiling facilitates diagnosis of pancreatic Malignancy. Sci Transl Med. 2017; 9:eaal3226. 10.1126/scitranslmed.aal3226 28539469 PMC5846089

[10] Yang Z, LaRiviere MJ, Ko J, Till JE, Christensen T, Yee SS, Black TA, Tien K, Lin A, Shen H, Bhagwat N, Herman D, Adallah A, et al. A multianalyte panel consisting of extracellular vesicle miRNAs and mRNAs, cfDNA, and CA19-9 shows utility for diagnosis and staging of pancreatic ductal adenocarcinoma. Clin Cancer Res. 2020; 26:3248–58. 10.1158/1078-0432.CCR-19-3313 32299821 PMC7334066

[11] Stroun M, Maurice P, Vasioukhin V, Lyautey J, Lederrey C, Lefort F, Rossier A, Chen XQ, Anker P. The origin and mechanism of circulating DNA. Ann N Y Acad Sci. 2000; 906:161–68. 10.1111/j.1749-6632.2000.tb06608.x 10818614

[12] De Rubis G, Rajeev Krishnan S, Bebawy M. Liquid biopsies in cancer diagnosis, monitoring, and prognosis. Trends Pharmacol Sci. 2019; 40:172–86. 10.1016/j.tips.2019.01.006 30736982

[13] Siravegna G, Marsoni S, Siena S, Bardelli A. Integrating liquid biopsies into the management of cancer. Nat Rev Clin Oncol. 2017; 14:531–48. 10.1038/nrclinonc.2017.14 28252003

[14] Wan JC, Massie C, Garcia-Corbacho J, Mouliere F, Brenton JD, Caldas C, Pacey S, Baird R, Rosenfeld N. Liquid biopsies come of age: towards implementation of circulating tumour DNA. Nat Rev Cancer. 2017; 17:223–38. 10.1038/nrc.2017.7 28233803

[15] Cohen JD, Javed AA, Thoburn C, Wong F, Tie J, Gibbs P, Schmidt CM, Yip-Schneider MT, Allen PJ, Schattner M, Brand RE, Singhi AD, Petersen GM, et al. Combined circulating tumor DNA and protein biomarker-based liquid biopsy for the earlier detection of pancreatic cancers. Proc Natl Acad Sci USA. 2017; 114:10202–07. 10.1073/pnas.1704961114 28874546 PMC5617273

[16] Samandari M, Julia MG, Rice A, Chronopoulos A, Del Rio Hernandez AE. Liquid biopsies for management of pancreatic cancer. Transl Res. 2018; 201:98–127. 10.1016/j.trsl.2018.07.008 30118658

[17] Lee JS, Park SS, Lee YK, Norton JA, Jeffrey SS. Liquid biopsy in pancreatic ductal adenocarcinoma: current status of circulating tumor cells and circulating tumor DNA. Mol Oncol. 2019; 13:1623–50. 10.1002/1878-0261.12537 31243883 PMC6670020

[18] Chen H, Tu H, Meng ZQ, Chen Z, Wang P, Liu LM. K-ras mutational status predicts poor prognosis in unresectable pancreatic cancer. Eur J Surg Oncol. 2010; 36:657–62. 10.1016/j.ejso.2010.05.014 20542658

[19] Cheng H, Liu C, Jiang J, Luo G, Lu Y, Jin K, Guo M, Zhang Z, Xu J, Liu L, Ni Q, Yu X. Analysis of ctDNA to predict prognosis and monitor treatment responses in metastatic pancreatic cancer patients. Int J Cancer. 2017; 140:2344–2350. 10.1002/ijc.30650 28205231

[20] Cheng H, Luo G, Jin K, Fan Z, Huang Q, Gong Y, Xu J, Yu X, Liu C. Kras mutation correlating with circulating regulatory T cells predicts the prognosis of advanced pancreatic cancer patients. Cancer Med. 2020; 9:2153–59. 10.1002/cam4.2895 32017404 PMC7064028

[21] Nakano Y, Kitago M, Matsuda S, Nakamura Y, Fujita Y, Imai S, Shinoda M, Yagi H, Abe Y, Hibi T, Fujii-Nishimura Y, Takeuchi A, Endo Y, et al. KRAS mutations in cell-free DNA from preoperative and postoperative sera as a pancreatic cancer marker: a retrospective study. Br J Cancer. 2018; 118:662–69. 10.1038/bjc.2017.479 29360815 PMC5846073

[22] Watanabe F, Suzuki K, Tamaki S, Abe I, Endo Y, Takayama Y, Ishikawa H, Kakizawa N, Saito M, Futsuhara K, Noda H, Konishi F, Rikiyama T. Longitudinal monitoring of KRAS-mutated circulating tumor DNA enables the prediction of prognosis and therapeutic responses in patients with pancreatic cancer. PLoS One. 2019; 14:e0227366. 10.1371/journal.pone.0227366 31891652 PMC6938323

[23] Castells A, Puig P, Móra J, Boadas J, Boix L, Urgell E, Solé M, Capellà G, Lluís F, Fernández-Cruz L, Navarro S, Farré A. K-ras mutations in DNA extracted from the plasma of patients with pancreatic carcinoma: diagnostic utility and prognostic significance. J Clin Oncol. 1999; 17:578–84. 10.1200/JCO.1999.17.2.578 10080602

[24] Earl J, Garcia-Nieto S, Martinez-Avila JC, Montans J, Sanjuanbenito A, Rodríguez-Garrote M, Lisa E, Mendía E, Lobo E, Malats N, Carrato A, Guillen-Ponce C. Circulating tumor cells (Ctc) and kras mutant circulating free Dna (cfdna) detection in peripheral blood as biomarkers in patients diagnosed with exocrine pancreatic cancer. BMC Cancer. 2015; 15:797. 10.1186/s12885-015-1779-7 26498594 PMC4619983

[25] Kinugasa H, Nouso K, Miyahara K, Morimoto Y, Dohi C, Tsutsumi K, Kato H, Matsubara T, Okada H, Yamamoto K. Detection of K-ras gene mutation by liquid biopsy in patients with pancreatic cancer. Cancer. 2015; 121:2271–80. 10.1002/cncr.29364 25823825

[26] Takai E, Totoki Y, Nakamura H, Morizane C, Nara S, Hama N, Suzuki M, Furukawa E, Kato M, Hayashi H, Kohno T, Ueno H, Shimada K, et al. Clinical utility of circulating tumor DNA for molecular assessment in pancreatic cancer. Sci Rep. 2015; 5:18425. 10.1038/srep18425 26669280 PMC4680882

[27] Hadano N, Murakami Y, Uemura K, Hashimoto Y, Kondo N, Nakagawa N, Sueda T, Hiyama E. Prognostic value of circulating tumour DNA in patients undergoing curative resection for pancreatic cancer. Br J Cancer. 2016; 115:59–65. 10.1038/bjc.2016.175 27280632 PMC4931379

[28] Tjensvoll K, Lapin M, Buhl T, Oltedal S, Steen-Ottosen Berry K, Gilje B, Søreide JA, Javle M, Nordgård O, Smaaland R. Clinical relevance of circulating KRAS mutated DNA in plasma from patients with advanced pancreatic cancer. Mol Oncol. 2016; 10:635–43. 10.1016/j.molonc.2015.11.012 26725968 PMC5423145

[29] Adamo P, Cowley CM, Neal CP, Mistry V, Page K, Dennison AR, Isherwood J, Hastings R, Luo J, Moore DA, Howard PJ, Miguel ML, Pritchard C, et al. Profiling tumour heterogeneity through circulating tumour DNA in patients with pancreatic cancer. Oncotarget. 2017; 8:87221–33. 10.18632/oncotarget.20250 29152076 PMC5675628

[30] Chen I, Raymond VM, Geis JA, Collisson EA, Jensen BV, Hermann KL, Erlander MG, Tempero M, Johansen JS. Ultrasensitive plasma ctDNA KRAS assay for detection, prognosis, and assessment of therapeutic response in patients with unresectable pancreatic ductal adenocarcinoma. Oncotarget. 2017; 8:97769–86. 10.18632/oncotarget.22080 29228650 PMC5716690

[31] Pietrasz D, Pécuchet N, Garlan F, Didelot A, Dubreuil O, Doat S, Imbert-Bismut F, Karoui M, Vaillant JC, Taly V, Laurent-Puig P, Bachet JB. Plasma Circulating Tumor DNA in Pancreatic Cancer Patients Is a Prognostic Marker. Clin Cancer Res. 2017; 23:116–123. 10.1158/1078-0432.CCR-16-0806 27993964

[32] Kim MK, Woo SM, Park B, Yoon KA, Kim YH, Joo J, Lee WJ, Han SS, Park SJ, Kong SY. Prognostic implications of multiplex detection of KRAS mutations in cell-free DNA from patients with pancreatic ductal adenocarcinoma. Clin Chem. 2018; 64:726–34. 10.1373/clinchem.2017.283721 29352043

[33] Lin M, Alnaggar M, Liang S, Chen J, Xu K, Dong S, Du D, Niu L. Circulating tumor DNA as a sensitive marker in patients undergoing irreversible electroporation for pancreatic cancer. Cell Physiol Biochem. 2018; 47:1556–64. 10.1159/000490874 29940591

[34] Perets R, Greenberg O, Shentzer T, Semenisty V, Epelbaum R, Bick T, Sarji S, Ben-Izhak O, Sabo E, Hershkovitz D. Mutant KRAS circulating tumor DNA is an accurate tool for pancreatic cancer monitoring. Oncologist. 2018; 23:566–72. 10.1634/theoncologist.2017-0467 29371474 PMC5947453

[35] Yang X, Xu W, Tian X, Wu J, Lv A, Li C, Guan X, Qian H, Hao C. Diagnostic and prognostic value of KRAS mutations in circulating pancreatic ductal adenocarcinoma tumor DNA. Translational Cancer Research. 2018; 7:622–33. 10.21037/tcr.2018.05.33

[36] Bernard V, Kim DU, San Lucas FA, Castillo J, Allenson K, Mulu FC, Stephens BM, Huang J, Semaan A, Guerrero PA, Kamyabi N, Zhao J, Hurd MW, et al. Circulating nucleic acids are associated with outcomes of patients with pancreatic cancer. Gastroenterology. 2019; 156:108–18.e4. 10.1053/j.gastro.2018.09.022 30240661 PMC6434712

[37] Groot VP, Mosier S, Javed AA, Teinor JA, Gemenetzis G, Ding D, Haley LM, Yu J, Burkhart RA, Hasanain A, Debeljak M, Kamiyama H, Narang A, et al. Circulating tumor DNA as a clinical test in resected pancreatic cancer. Clin Cancer Res. 2019; 25:4973–84. 10.1158/1078-0432.CCR-19-0197 31142500 PMC7403524

[38] Lee B, Lipton L, Cohen J, Tie J, Javed AA, Li L, Goldstein D, Burge M, Cooray P, Nagrial A, Tebbutt NC, Thomson B, Nikfarjam M, et al. Circulating tumor DNA as a potential marker of adjuvant chemotherapy benefit following surgery for localized pancreatic cancer. Ann Oncol. 2019; 30:1472–78. 10.1093/annonc/mdz200 31250894 PMC6771221

[39] Mohan S, Ayub M, Rothwell DG, Gulati S, Kilerci B, Hollebecque A, Sun Leong H, Smith NK, Sahoo S, Descamps T, Zhou C, Hubner RA, McNamara MG, et al. Analysis of circulating cell-free DNA identifies KRAS copy number gain and mutation as a novel prognostic marker in pancreatic cancer. Sci Rep. 2019; 9:11610. 10.1038/s41598-019-47489-7 31406261 PMC6690979

[40] Patel H, Okamura R, Fanta P, Patel C, Lanman RB, Raymond VM, Kato S, Kurzrock R. Clinical correlates of blood-derived circulating tumor DNA in pancreatic cancer. J Hematol Oncol. 2019; 12:130. 10.1186/s13045-019-0824-4 31801585 PMC6894333

[41] Strijker M, Soer EC, de Pastena M, Creemers A, Balduzzi A, Beagan JJ, Busch OR, van Delden OM, Halfwerk H, van Hooft JE, van Lienden KP, Marchegiani G, Meijer SL, et al. Circulating tumor DNA quantity is related to tumor volume and both predict survival in metastatic pancreatic ductal adenocarcinoma. Int J Cancer. 2020; 146:1445–56. 10.1002/ijc.32586 31340061 PMC7004068

[42] Guo S, Shi X, Shen J, Gao S, Wang H, Shen S, Pan Y, Li B, Xu X, Shao Z, Jin G. Preoperative detection of KRAS G12D mutation in ctDNA is a powerful predictor for early recurrence of resectable PDAC patients. Br J Cancer. 2020; 122:857–67. 10.1038/s41416-019-0704-2 31969677 PMC7078253

[43] Schlitter AM, Segler A, Steiger K, Michalski CW, Jäger C, Konukiewitz B, Pfarr N, Endris V, Bettstetter M, Kong B, Regel I, Kleeff J, Klöppel G, Esposito I. Molecular, morphological and survival analysis of 177 resected pancreatic ductal adenocarcinomas (PDACs): identification of prognostic subtypes. Sci Rep. 2017; 7:41064. 10.1038/srep41064 28145465 PMC5286512

[44] Balaban EP, Mangu PB, Khorana AA, Shah MA, Mukherjee S, Crane CH, Javle MM, Eads JR, Allen P, Ko AH, Engebretson A, Herman JM, Strickler JH, Benson AB 3rd, Urba S, Yee NS. Locally Advanced, Unresectable Pancreatic Cancer: American Society of Clinical Oncology Clinical Practice Guideline. J Clin Oncol. 2016; 34:2654–68. 10.1200/JCO.2016.67.5561 27247216

[45] Sohal DP, Kennedy EB, Khorana A, Copur MS, Crane CH, Garrido-Laguna I, Krishnamurthi S, Moravek C, O’Reilly EM, Philip PA, Ramanathan RK, Ruggiero JT, Shah MA, et al. Metastatic pancreatic cancer: ASCO clinical practice guideline update. J Clin Oncol. 2018; 36:2545–56. 10.1200/JCO.2018.78.9636 29791286 PMC7504972

[46] Khorana AA, McKernin SE, Berlin J, Hong TS, Maitra A, Moravek C, Mumber M, Schulick R, Zeh HJ, Katz MH. Potentially curable pancreatic adenocarcinoma: ASCO clinical practice guideline update. J Clin Oncol. 2019; 37:2082–88. 10.1200/JCO.19.00946 31180816

[47] Merker JD, Oxnard GR, Compton C, Diehn M, Hurley P, Lazar AJ, Lindeman N, Lockwood CM, Rai AJ, Schilsky RL, Tsimberidou AM, Vasalos P, Billman BL, et al. Circulating Tumor DNA Analysis in Patients With Cancer: American Society of Clinical Oncology and College of American Pathologists Joint Review. J Clin Oncol. 2018; 36:1631–1641. 10.1200/JCO.2017.76.8671 29504847

[48] Yoon C, Till J, Cho SJ, Chang KK, Lin JX, Huang CM, Ryeom S, Yoon SS. KRAS activation in gastric adenocarcinoma stimulates epithelial-to-mesenchymal transition to cancer stem-like cells and promotes metastasis. Mol Cancer Res. 2019; 17:1945–57. 10.1158/1541-7786.MCR-19-0077 31217166 PMC6726517

[49] Henriksen SD, Madsen PH, Larsen AC, Johansen MB, Pedersen IS, Krarup H, Thorlacius-Ussing O. Cell-free DNA promoter hypermethylation in plasma as a predictive marker for survival of patients with pancreatic adenocarcinoma. Oncotarget. 2017; 8:93942–56. 10.18632/oncotarget.21397 29212200 PMC5706846

[50] Moher D, Liberati A, Tetzlaff J, Altman DG, and PRISMA Group. Preferred reporting items for systematic reviews and meta-analyses: the PRISMA statement. Int J Surg. 2010; 8:336–41. 10.1016/j.ijsu.2010.02.007 20171303

[51] Parmar MK, Torri V, Stewart L. Extracting summary statistics to perform meta-analyses of the published literature for survival endpoints. Stat Med. 1998; 17:2815–34. 10.1002/(sici)1097-0258(19981230)17:24<2815::aid-sim110>3.0.co;2-8 9921604

[52] Tierney JF, Stewart LA, Ghersi D, Burdett S, Sydes MR. Practical methods for incorporating summary time-to-event data into meta-analysis. Trials. 2007; 8:16. 10.1186/1745-6215-8-16 17555582 PMC1920534

[53] Margulis AV, Pladevall M, Riera-Guardia N, Varas-Lorenzo C, Hazell L, Berkman ND, Viswanathan M, Perez-Gutthann S. Quality assessment of observational studies in a drug-safety systematic review, comparison of two tools: the newcastle-ottawa scale and the RTI item bank. Clin Epidemiol. 2014; 6:359–68. 10.2147/CLEP.S66677 25336990 PMC4199858

