# Supplementary material for: Prognostic value of circulating tumor DNA in pancreatic cancer: a systematic review and meta-analysis
Source: Aging (Albany NY). 2020 Dec 9;13(2):2031–48. doi: 10.18632/aging.202199 (PMC7880399; doi:10.18632/aging.202199)
Supplement: Supplementary Figure 1 [file aging-13-202199-s001.pdf]

SUPPLEMENTARY FIGURE

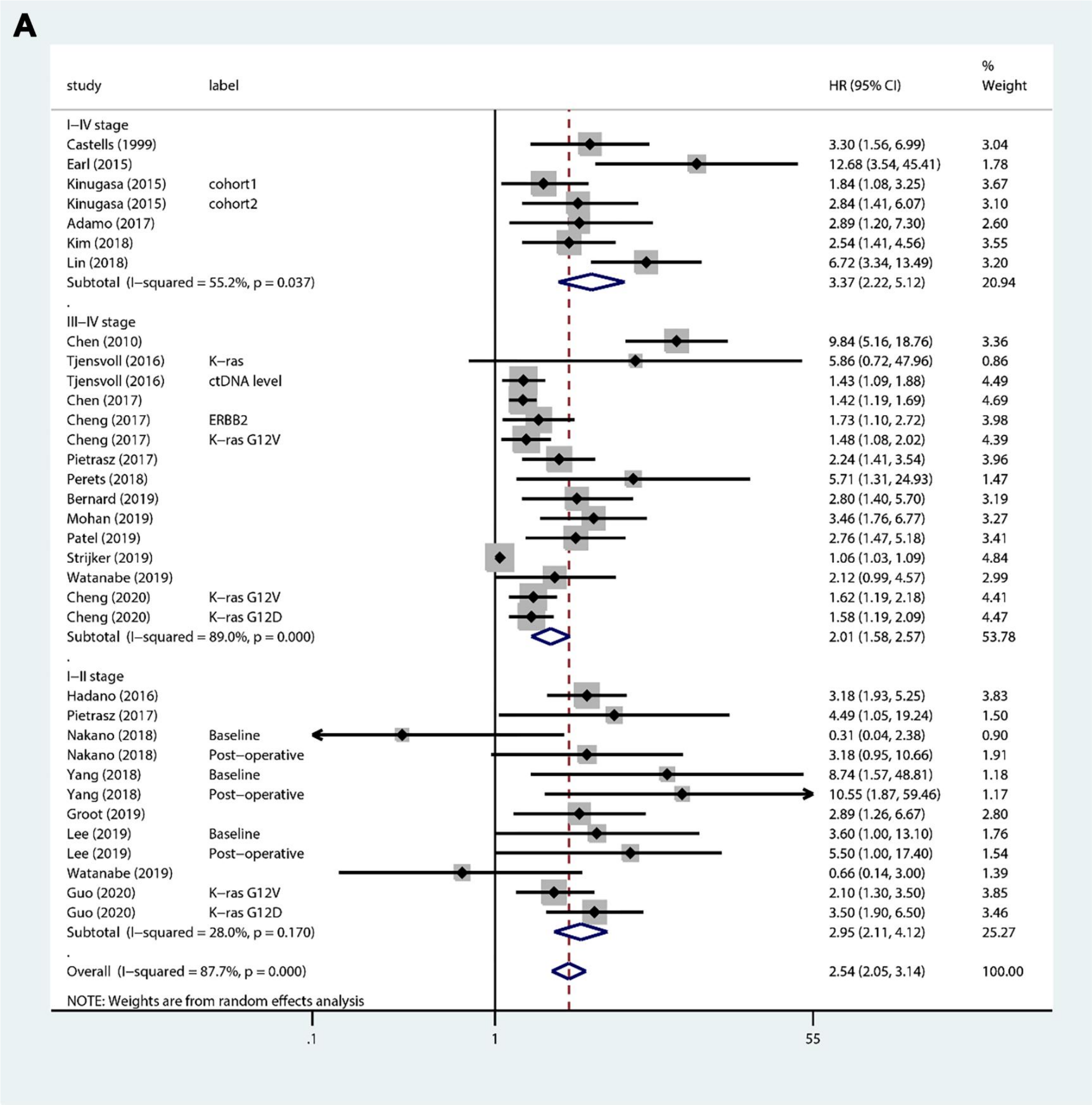

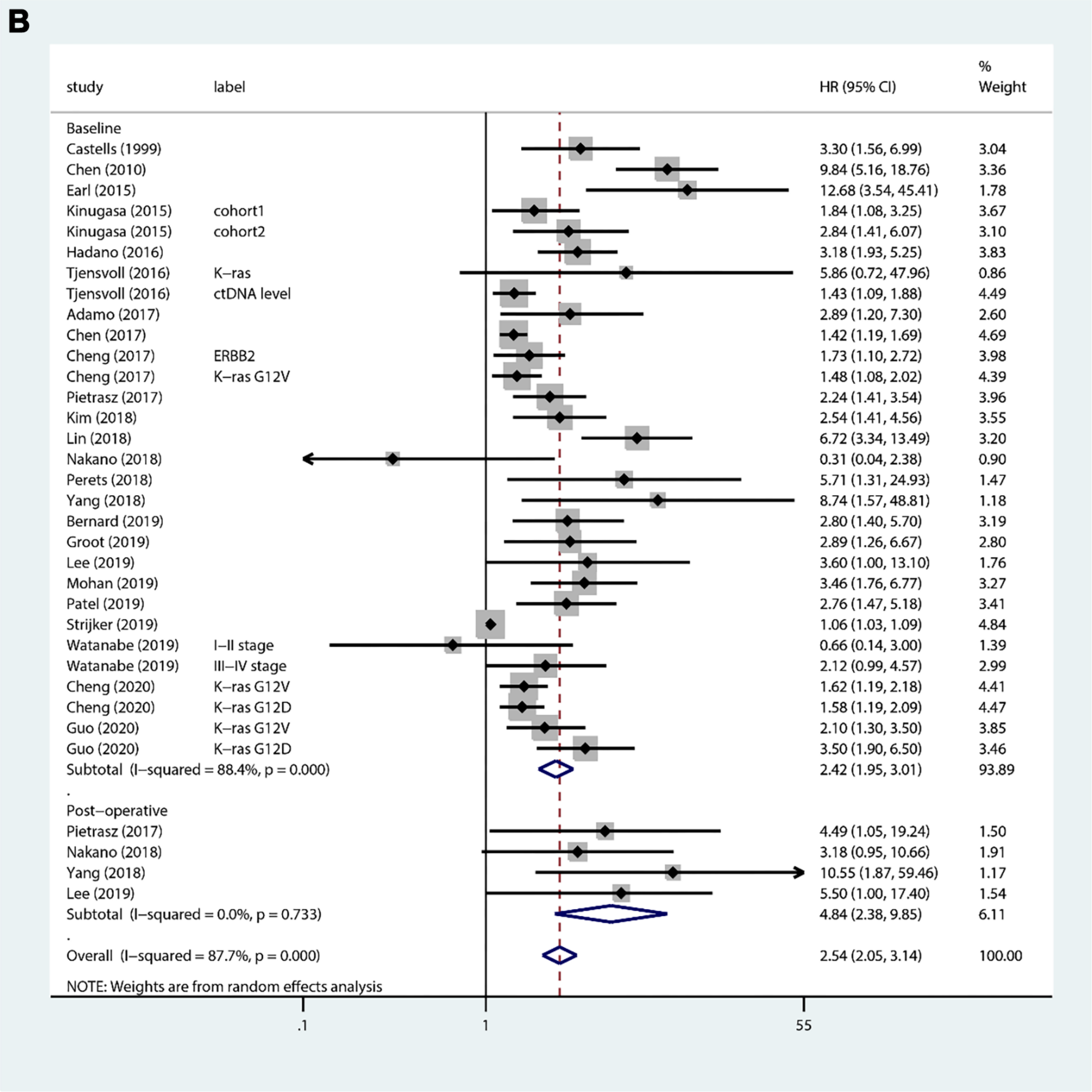
B
study
label
HR (95% CI)
%
Weight
Baseline
Castells (1999) 3.30 (1.56, 6.99) 3.04
Chen (2010) 9.84 (5.16, 18.76) 3.36
Earl (2015) 12.68 (3.54, 45.41) 1.78
Kinugasa (2015) cohort1 1.84 (1.08, 3.25) 3.67
Kinugasa (2015) cohort2 2.84 (1.41, 6.07) 3.10
Hadano (2016) 3.18 (1.93, 5.25) 3.83
Tjensvoll (2016) K–ras 5.86 (0.72, 47.96) 0.86
Tjensvoll (2016) ctDNA level 1.43 (1.09, 1.88) 4.49
Adamo (2017) 2.89 (1.20, 7.30) 2.60
Chen (2017) 1.42 (1.19, 1.69) 4.69
Cheng (2017) ERBB2 1.73 (1.10, 2.72) 3.98
Cheng (2017) K–ras G12V 1.48 (1.08, 2.02) 4.39
Pietrasz (2017) 2.24 (1.41, 3.54) 3.96
Kim (2018) 2.54 (1.41, 4.56) 3.55
Lin (2018) 6.72 (3.34, 13.49) 3.20
Nakano (2018) 0.31 (0.04, 2.38) 0.90
Perets (2018) 5.71 (1.31, 24.93) 1.47
Yang (2018) 8.74 (1.57, 48.81) 1.18
Bernard (2019) 2.80 (1.40, 5.70) 3.19
Groot (2019) 2.89 (1.26, 6.67) 2.80
Lee (2019) 3.60 (1.00, 13.10) 1.76
Mohan (2019) 3.46 (1.76, 6.77) 3.27
Patel (2019) 2.76 (1.47, 5.18) 3.41
Strijker (2019) 1.06 (1.03, 1.09) 4.84
Watanabe (2019) I–II stage 0.66 (0.14, 3.00) 1.39
Watanabe (2019) III–IV stage 2.12 (0.99, 4.57) 2.99
Cheng (2020) K–ras G12V 1.62 (1.19, 2.18) 4.41
Cheng (2020) K–ras G12D 1.58 (1.19, 2.09) 4.47
Guo (2020) K–ras G12V 2.10 (1.30, 3.50) 3.85
Guo (2020) K–ras G12D 3.50 (1.90, 6.50) 3.46
Subtotal (I–squared = 88.4%, p = 0.000) 2.42 (1.95, 3.01) 93.89
.
Post–operative
Pietrasz (2017) 4.49 (1.05, 19.24) 1.50
Nakano (2018) 3.18 (0.95, 10.66) 1.91
Yang (2018) 10.55 (1.87, 59.46) 1.17
Lee (2019) 5.50 (1.00, 17.40) 1.54
Subtotal (I–squared = 0.0%, p = 0.733) 4.84 (2.38, 9.85) 6.11
.
Overall (I–squared = 87.7%, p = 0.000) 2.54 (2.05, 3.14) 100.00
NOTE: Weights are from random effects analysis
.1
1
55

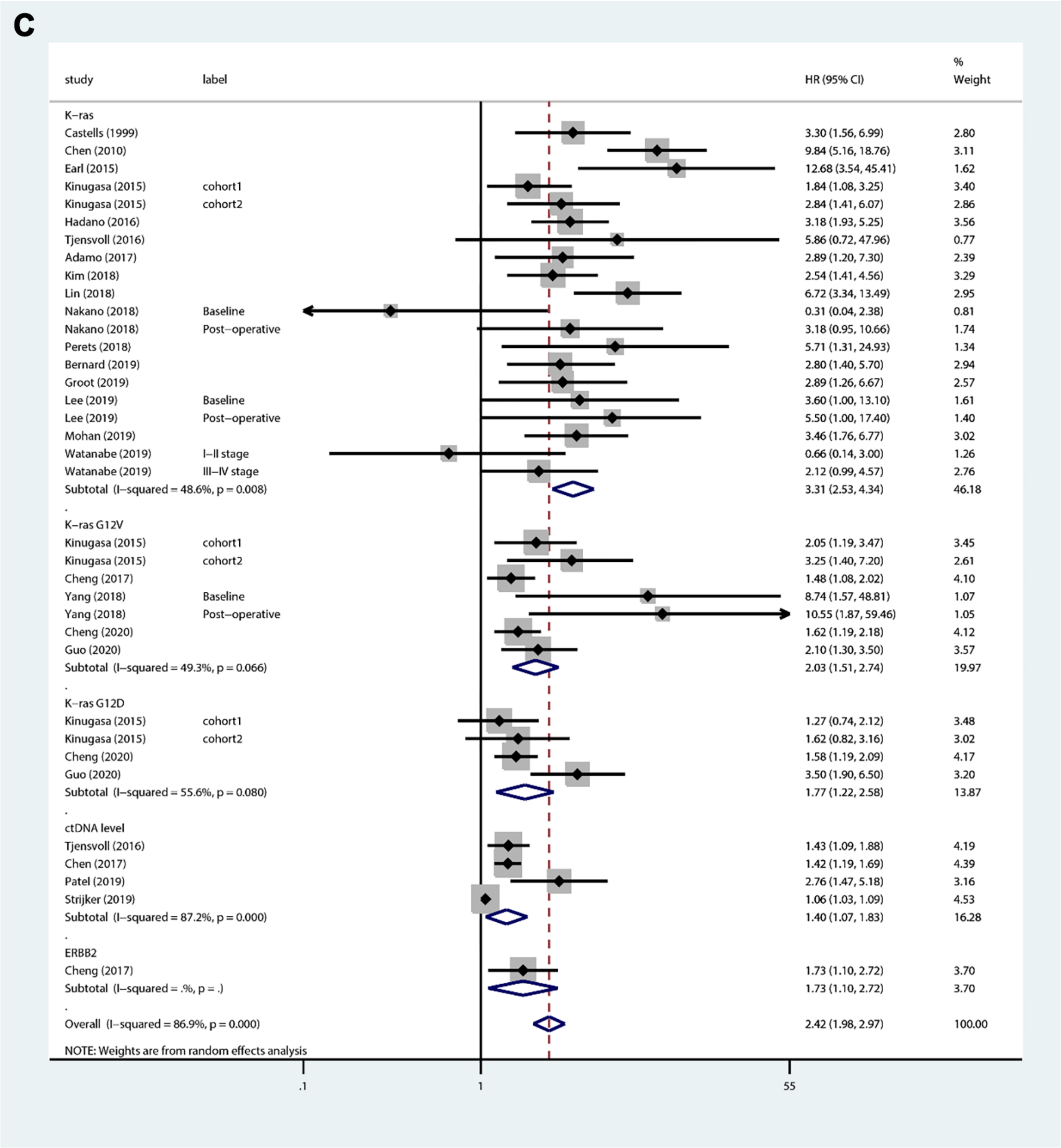

Supplementary Figure 1. (**A**) Subgroup analyses of OS according to tumor stage. (**B**) Subgroup analyses of OS according to sampling time. (**C**) Subgroup analyses of OS according to detection marker.
