# Supplementary material for: Prognostic value of circulating tumor DNA in pancreatic cancer: a systematic review and meta-analysis
Source: Aging (Albany NY). 2020 Dec 9;13(2):2031–48. doi: 10.18632/aging.202199 (PMC7880399; doi:10.18632/aging.202199)
Supplement: Supplementary Table 1 [file aging-13-202199-s002.pdf]

SUPPLEMENTARY TABLE

Supplementary Table 1. Quality assessment of the included studies with Newcastle-Ottawa Scale (NOS).

	Selection					Outcome			
**Study**	**Representativeness of the exposed cohort**	**Selection of the non-exposed cohort**	**Ascertainment of exposure**	**Demonstration that outcome of interest was not present at start of study**	**Comparability**	**Assessment of outcome**	**Was follow-up long enough for outcomes to occur**	**Adequacy of follow-up of cohorts**	**Overall**
Castells 1999	☆	☆	☆	☆	☆	☆		☆	7
Chen 2010	☆	☆	☆	☆	☆☆	☆			7
Earl 2015	☆	☆	☆	☆	☆	☆	☆		7
Kinugasa 2015	☆	☆	☆	☆	☆	☆		☆	7
Takai 2015	☆	☆	☆	☆	☆	☆			6
Hadano 2016	☆	☆	☆	☆	☆☆	☆	☆	☆	9
Tjensvoll 2016	☆	☆	☆	☆	☆☆	☆			7
Adamo 2017	☆	☆	☆	☆	☆	☆	☆	☆	8
Chen 2017	☆	☆	☆	☆	☆	☆	☆	☆	8
Cheng 2017	☆	☆	☆	☆	☆☆	☆	☆	☆	9
Pietrasz 2017	☆	☆	☆	☆	☆☆	☆	☆		8
Kim 2018	☆	☆	☆	☆	☆	☆			6
Lin 2018	☆	☆	☆	☆	☆	☆		☆	7
Nakano 2018	☆	☆	☆	☆	☆☆	☆	☆		8
Perets 2018	☆	☆	☆	☆	☆	☆	☆	☆	9
Yang 2018	☆	☆	☆	☆	☆☆	☆			7
Bernard 2019	☆	☆	☆	☆	☆☆	☆	☆		8
Groot 2019	☆	☆	☆	☆	☆☆	☆	☆		8
Lee 2019	☆	☆	☆	☆	☆☆	☆	☆		8
Mohan 2019	☆	☆	☆	☆	☆	☆	☆	☆	8
Patel 2019	☆	☆	☆	☆	☆☆	☆	☆		8
Strijker 2019	☆	☆	☆	☆	☆	☆		☆	7
Watanabe 2019	☆	☆	☆	☆	☆	☆	☆		7
Cheng 2020	☆	☆	☆	☆	☆	☆	☆	☆	8
Guo 2020	☆	☆	☆	☆	☆☆	☆	☆	☆	9
